# Phylogeography of social polymorphism in a boreo-montane ant

**DOI:** 10.1186/s12862-016-0711-3

**Published:** 2016-06-23

**Authors:** Jürgen Trettin, Shobhit Agrawal, Jürgen Heinze

**Affiliations:** Zoology / Evolutionary Biology, Universität Regensburg, 93040 Regensburg, Germany; Alfred Wegener Institute Helmholtz Centre for Polar and Marine Research, 27570 Bremerhaven, Germany

**Keywords:** Bottlenecks, Extra-Mediterranean refugia, Gene flow, *Leptothorax acervorum*, Phylogeography, Plastic behaviour, Population structure, Quaternary climate change, Reproductive skew

## Abstract

**Background:**

The disjunct distribution of several Palearctic species has been widely shaped by the changes in climatic conditions during the Quaternary. The observed genetic differentiation or reproductive isolation between extant populations may be the outcome of their contemporary geographic separation or reproductive incompatibility due to differences in phenotypic traits which have evolved in isolated refugia. In the boreal ant *Leptothorax acervorum*, colonies from central and peripheral populations differ in social structure: colonies from Central and Northern Europe may contain several equally reproductive queens (facultative polygyny), while in colonies from peripheral populations in Spain only one the most dominant of several queens lays eggs (functional monogyny). By reconstructing the specie’s evolutionary and demographic history in Southwestern Europe we examine whether variation in social organization is associated with restricted gene flow between the two social forms.

**Results:**

We show that multi-queen colonies from all so far known inner Iberian populations of *L. acervorum* are functionally monogynous, whereas multi-queen colonies from all Pyrenean populations are polygynous, like those from other previously studied areas in Europe. Our analyses revealed complex spatial-genetic structure, but no association between spatial-genetic structure and social organization in SW-Europe. The population in the western Pyrenees diverged most strongly from other Iberian populations. Moreover, microsatellite data suggest the occurrence of recent bottlenecks in Pyrenean and inner Iberian populations.

**Conclusions:**

Our study shows a lack of reproductive isolation between the two social forms in SW-Europe. This in turn suggests that demographic and spatial patterns in genetic variation as well as the distribution of social phenotypes are better explained by co-variation with climatic, ecological, and historical factors. Moreover, we for the first time show the existence of substantial spatial-genetic structure in *L. acervorum*, suggesting the existence of multiple refugia in SW-Europe, including two extra-Mediterranean refugia in France. While gene flow among inner Iberian refugia may have been larger during the late glacial, extra-Mediterranean refugia in southern France may have contributed to the post-glacial recolonization of W-Europe.

**Electronic supplementary material:**

The online version of this article (doi:10.1186/s12862-016-0711-3) contains supplementary material, which is available to authorized users.

## Background

Quaternary climate changes and their dramatic effects on demography and distributional ranges have left behind their footprints on the evolution of Palearctic biota. Temperate species escaped the last glacial maximum (LGM 23-18 ka BP) by retreating southwards to suitable habitats in Iberia, Italy and Balkans and recolonized Northern and Central Europe from these refugia after the retreat of the ice sheet [1, 2]. In contrast, boreal species could survive in periglacial areas in Central Europe and expanded their ranges into more southern areas. During postglacial warming, southern populations of boreal species often became “trapped” in mountainous regions, leading to their present arctic-alpine or boreo-montane distribution [3, 4].

Relict populations of boreal species in Mediterranean refugia may be exposed to other environmental conditions than populations from the centre of their range and thus accumulate unique genotypes and specific adaptations [5, 6]. This is particularly evident in the boreo-montane ant *Leptothorax acervorum* (Fabricius, 1793) (Hymenoptera, Formicidae). Across its range, a large fraction of *L. acervorum* colonies contain several reproductive queens (the others are queenless or have a single queen). Interestingly, the partitioning of reproduction among nestmate queens in such multi-queen colonies (“reproductive skew”) varies among populations [7, 8]. In colonies from boreal and temperate Eurasia, all queens contribute equally to the brood (low skew, “facultative polygyny”, [9]). In contrast, queens in colonies from the range margin (e.g. Central Spain and Hokkaido, Japan) form social and reproductive hierarchies and only the top-ranking queen reproduces (high skew, “functional monogyny”, [8, 10, 11]). In addition, mating behaviour differs between populations from Central Spain and Central Europe [8].

From these pronounced behavioural differences we expected to find genetic differentiation with reproductive isolation and incipient speciation between the two social forms. Alternatively, a lack of congruence between spatial patterns of genetic structure and social behaviour would indicate environment-induced phenotypic plasticity underlying geographic variation in behaviour (e.g. [12–15]).

The present distribution of *L. acervorum* reaches as far north as the tundra-taiga ecotone at the North Cape and the Lena delta [16, 17], suggesting that it was capable of surviving the ice ages in refugia in Central Europe. This should have left conspicuous traces on the genetic structure and diversity of extant populations, whose reconstruction should result in a better understanding of the respective contributions of potential extra-Mediterranean and “classical” southern-European refugia towards the current distribution of genetic diversity of W-Palaearctic populations of *L. acervorum*.

The aims of this study, therefore, were (i) to map the distribution of the two social phenotypes of *L. acervorum* in inner Iberian mountains and Pyrenees, (ii) to analyse the micro-evolutionary history of the two social forms using genetic markers, and (iii) to infer the population history of *L. acervorum* in SW-Europe in a broader phylogeographic context.

## Methods

### Ant sampling, DNA extraction and queen ovary dissection

**Fig. 1:**
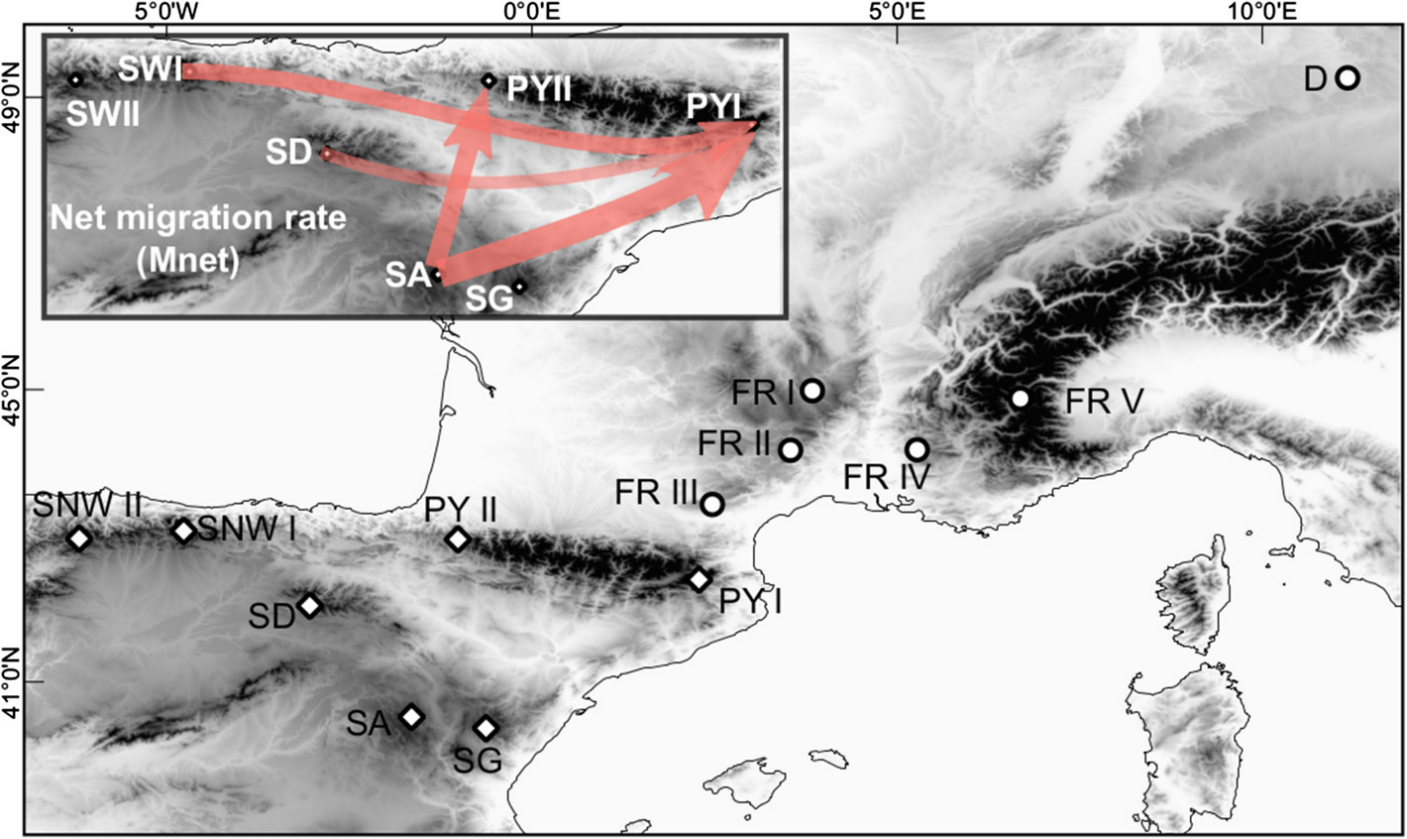
Map showing sampling locations of *L. acervorum* in SW- and C-Europe (for details on locations see Table 1). Elevation levels (m a. s. l.) are given in different shades (*black*: above 1500, *dark grey*: 1000–1500, grey: 500–1000, *light grey*: 0–500). *Circles*: refer to mtDNA-only samples. Diamonds: refer to locations sampled for mtDNA and microsatellites. Inlay shows net migration between inner Iberian and Pyrenean populations (estimated from microsatellites)

Colonies of *Leptothorax acervorum* were collected between 2008 and 2011 in the Iberian Peninsula (SG, SA, SD, SNW I, SNWII), the Pyrenees (PY I, PY II), southern France (FR I to V) and Germany (D) (see Table 1 & Fig. 1 for details on locations and abbreviations).

**Table 1:** Sampling information for the study area of *Leptothorax acervorum* in SW-Europe, Germany and England

ID	Population name	Region	Location (N, E)	Year	No. of samples (STR/mtDNA)
PY I	eastern Pyrenees	Pyrenees	42.401°, 2.288°	2009	15 / 14
PY II	western Pyrenees	Pyrenees	42.953°, −1.012°	2009	15 / 3
SNW I	Cantabrian Mts. I	Inner Iberia	43.067°, −4.766°	2010	15 / 4
SNW II	Cantabrian Mts. II	Inner Iberia	42.963°, −6.197°	2010	15 / 3
SG	Sierra de Gúdar	Inner Iberia	40.371°, −0.627°	2009	15 / 3
SA	Sierra de Albarracin	Inner Iberia	40.525°, −1.647°	2008, 2009, 2010	15 / 3
SD	Sierra de la Demanda	Inner Iberia	42.044°, −3.040°	2009, 2010	15 / 3
FR I	Massif Central I	France	44.985°, 3.840°	2011	* / 2
FR II	Massif Central II	France	44.176°, 3.541°	2011	* / 6
FR III	Massif Central III	France	43.433°, 2.471°	2011	* / 3
FR IV	Mont Ventoux	France	44.182°, 5.275°	2011	* / 5
FR V	Western Alps	France	44.877°, 6.688°	2011	* / 3
D	Germany	Central Europe	49.266°, 11.168°	2011	* / 8
E	England	Central Europe	^***§***^	^***§***^	* / 2

Population ID, geographic location, collection year, geographic region and number of samples per population. * Populations not genotyped for microsatellites (STR). ^***§***^ See [18] for details

One worker each from 105 colonies from the Iberian Peninsula and the Pyrenees was genotyped at ten polymorphic microsatellite loci. The mitochondrial DNA dataset contains sequences of 62 workers representing all sampled locations and geographic regions. In addition, we used DNA-samples from England (E, see [18] and Table 1). All specimens were preserved in 95 % ethanol or frozen at −20 °C until DNA extraction. Genomic DNA was extracted from whole workers using a cetylmethyl ammonium bromide (CTAB)-protocol [19].

Additionally, for the determination of social structure and reproductive skew we examined how many of the queens co-occurring in single multi-queen nests (a colony) had been reproductively active by dissecting their ovaries (528 queens from 66 colonies from all over Pyrenees (PY) and four inner Iberian populations (SG, SD, SNW I, SNW II). From the 12 Pyrenean colonies one was collected from PY I and four from PY II. The remaining seven colonies were collected from three additional Pyrenean locations (no. of colonies per location; 1, 2, and 4, respectively). Previous analysis had already confirmed functional monogyny for the SA population [8, 20]. Ovary dissections were conducted right after transfer of colonies to the laboratory (August 2009: SG; November 2009: PY, SD; February 2010: SD; July 2010: SNW I, SNW II, SD). For further details on ovary dissections and determination of reproductive skew see Additional file 1.

### Microsatellite analyses

Four of the ten used microsatellite loci were developed specifically for *L. acervorum* (GA1, GA2, GT1, GT2; [21]. The remaining loci represent cross-amplifications from other ants (GT218, GT223; [22], 2MS67; [23], 2MS46; Suefuji et al., unpublished – 2MS46fwd: 5′- GCTCACTACTATGCTGCCAGC -3′, 2MS46rev: 5′- TCCGTCTATCCCTTCCTGCAA -3′, L18; [24] and Myrt3; [25]. PCR conditions were chosen as follows: initial denaturation 5 min at 95 °C; 35 cycles of 60 s at 95 °C, 45 s at the locus-specific annealing temperature of 45–60 °C, elongation of 45 s at 72 °C and a locus-specific final extension step of 30–120 s at 72 °C. Total reaction volume was 20 μL with 1 μL DNA template. PCR products were either analysed on an ABI PRISM 310 automated sequencer (GA1, GA2, GT218, GT223) and subsequently genotyped using GENESCAN 3.1 (Applied Biosystems) or sent to GATC Biotech AG (GT1, GT2, L18, Myrt3a, 2MS67, 2MS46 (II)) and subsequently genotyped using Peak Scanner Software v1.0 (Applied Biosystems).

**Fig. 2:**
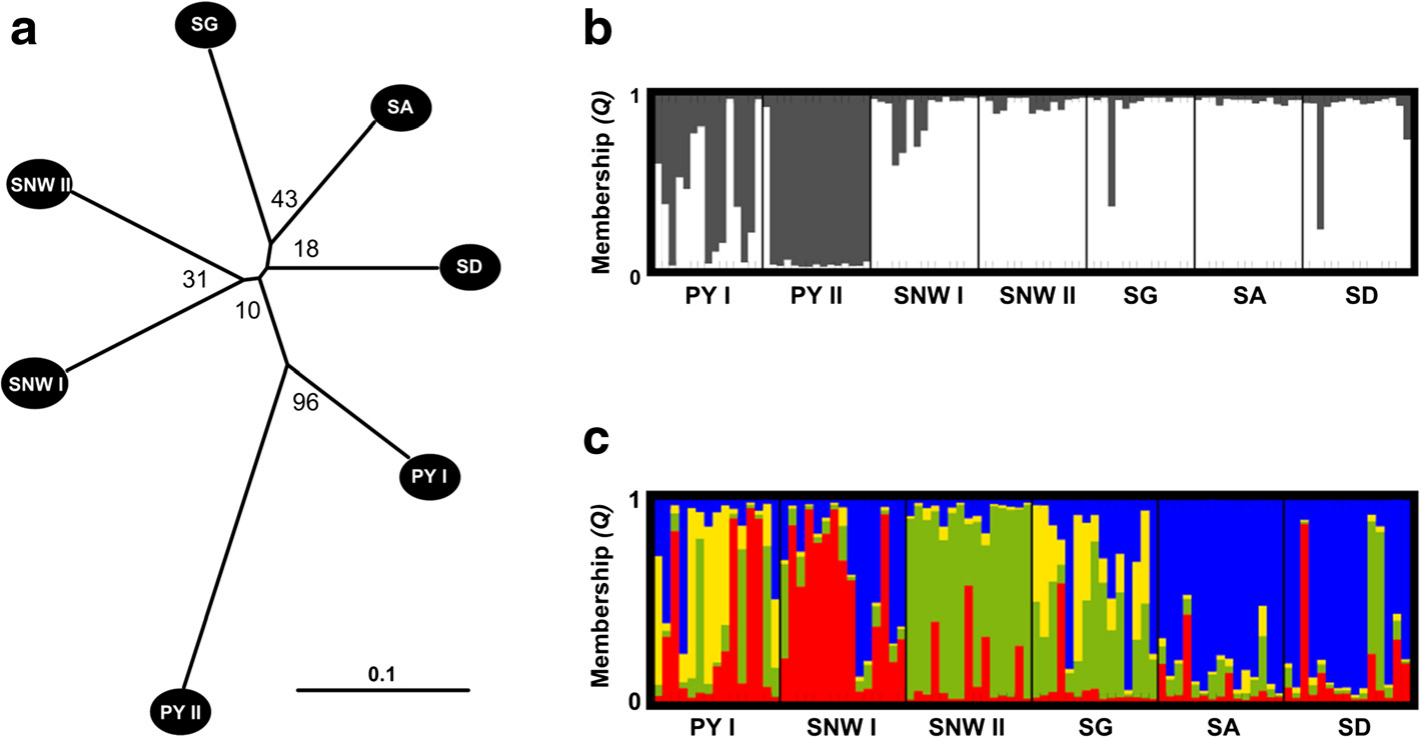
Analysis of genetic structure and relationship between and within Iberian populations (for microsatellites) of *L. acervorum*. **a** Unrooted neighbour-joining tree using Nei's *D*_*A*_ distance (bootstrap values given as numbers close to nodes). **b** Bayesian assignment analysis with all samples included (individual membership proportions for *K* = 2 clusters). **c** Bayesian assignment analysis without samples from PY II (individual membership proportions for *K* = 4 clusters)

Each microsatellite locus was tested for deviation from Hardy-Weinberg equilibrium (HWE) using exact HW tests [26]. For testing of independence between loci, we assessed linkage disequilibrium (LD) for all locus pairs across populations (Fisher’s method). Both tests were run in GENEPOP 4.2.2 [27]. Evidence for the occurrence of null alleles was assessed and corrected using MICRO-CHECKER 2.2.3 [28]. We found evidence for null alleles in 14 of out of 70 microsatellite loci x population pairs, of which only three pairs had null allele frequencies larger than 0.2 (maximum: 0.243, see Additional file 2). Subsequent analysis of both datasets (original and null allele corrected) resulted only in marginal differences in global and pairwise *F*_*ST*_ – values and microsatellite-tree topologies (null allele corrected global *F*_*ST*_ = 0.074 and see Fig. 2a, Table 5 and Additional file 2). We, therefore, decided to perform all downstream analyses with the original microsatellite dataset if not explicitly stated otherwise.

#### Population structure, gene flow and demographic history

Number of alleles (*k*), allelic richness (*A*), number of private alleles (*A*_*P*_), and expected (H_E_) and observed (H_O_) heterozygosities were calculated per population and locus using FSTAT 2.9.3.2 [29] and GENALEX 6.5 [30]. The genealogical relationships among study populations were analysed by neighbour-joining trees in POPULATIONS 1.2.31 [31] using D_*A*_ distance [32]. Bootstrap values were obtained by 2000 replications over loci. To study population structure in more detail we estimated overall and pairwise F-statistics using GENEPOP as well as GENALEX to calculate allelic-diversity corrected *F*_*ST*_ – value analogues (Hedrick’s standardized *G*_*ST*_, *G*_*ST*_ corrected for small sample sizes *G”*_*ST*_, and Jost’s *D,* [33]). To test the hypothesis that genetic differentiation is equal or higher within than between regions (defined here as geographic regions – Iberian Peninsula (IB) and Pyrenees (PY) or high skew vs. low skew populations), we conducted an analysis of molecular variance (AMOVA) using ARLEQUIN v3.5.1.2 [34]. Significance of results was evaluated over 10,000 replicates.

We applied Bayesian clustering to assess fine-scale population genetic structure (as well as identifying distinct populations) based upon multi-locus genotypic data. Cluster analysis was run in STRUCTURE 2.3.4 [35] allowing individuals to have mixed ancestry (admixture model) without using sampling locations as prior information and without correlated allele frequencies among populations. As recommended by ([35], and cf. STRUCTURE documentation), we run a first analysis including microsatellite data from all locations and in a second analysis excluded samples from the most divergent population (PY II). Potential population cluster values (*K*) varied from 1 to 10 with ten runs per value of *K*, burn-in and sampling period were set to 300,000 (first analysis) and to 200,000 generations (second analysis) accordingly. For each analysis the optimal *K* value was assessed by following the ΔK-method as described by [36] and implemented in STRUCTURE HARVESTER web-v0.6.93 [37]. DISTRUCT v1.1 [38] was used to graphically display the output. Additionally, we used a Mantel test [39] to evaluate genetic isolation by distance in the microsatellite data, with significance of results evaluated by 999 matrix permutations in GENALEX. Genetic distance matrix among populations was calculated as linearized *F*_ST_-values [(*F*_ST_ (1- *F*_ST_)^−1^] in FSTAT whereas geographical distances among populations in kilometres were calculated in GENALEX (based upon the coordinates shown in Table 1).

Because of the patchy distribution of *L. acervorum* in “mountainous islands” in Central Spain, we tested for the occurrence of bottlenecks in each sampled population. First, we used Wilcoxon’s sign rank test, which tests for an excess of heterozygosity, implemented in BOTTLENECK 1.2.02 [40]. Second, we calculated the *M*-ratio statistic [41] to test for a severe reduction in effective population size. Finally, we used the coalescent-based Bayesian MCMC algorithm as implemented in MIGRATE-N v 3.6.10 [42] for the inference of demographic parameters, the analyses of demographic changes over time and estimation of pairwise migration rates. For details on all methods (including parameter settings) see Additional file 1.

### Mitochondrial DNA analyses

The primers C1-J-2183 and A8-N-3914 [43] were used to amplify an 1641 bp long mitochondrial DNA fragment, starting from within the COI gene, including the complete COII sequence, and finishing in the very beginning of the ATPase 8 gene. PCR was carried out in a total reaction volume of 15 μL using the BIO-X-ACT Short Mix (Bioline) and 1 μL DNA template. PCR conditions consisted of an initial denaturation 4 min at 94 °C; 38 cycles of 75 s at 94 °C, 75 s at 50 °C (annealing), elongation of 150 s at 72 °C, and a final extension step of 5 min at 72 °C. PCR products were sent to LGC Genomics for purification and Sanger sequencing.

Chromatograms were assessed and edited using CHROMAS LITE 2.1.1 (Technelysium) and subsequently concatenated in BIOEDIT [44]. Sequences were aligned manually and automatically by using the algorithm CLUSTAL W as implemented in BIOEDIT. All sequences could be aligned unambiguously, and no indels were found except in three sequences from the eastern Pyrenees (PY I) that contained a 1 bp-deletion. Since this appeared in a noncoding region only, it was considered valid and was used as a fifth mutational state in network analysis. The absence of unusual stop codons as potential evidence for the presence of pseudogenes (numts, e.g. [45]) was checked in the ARTEMIS GENOME BROWSER using the invertebrate mitochondrial codon table [46].

#### Phylogeography and population structure

**Fig. 3:**
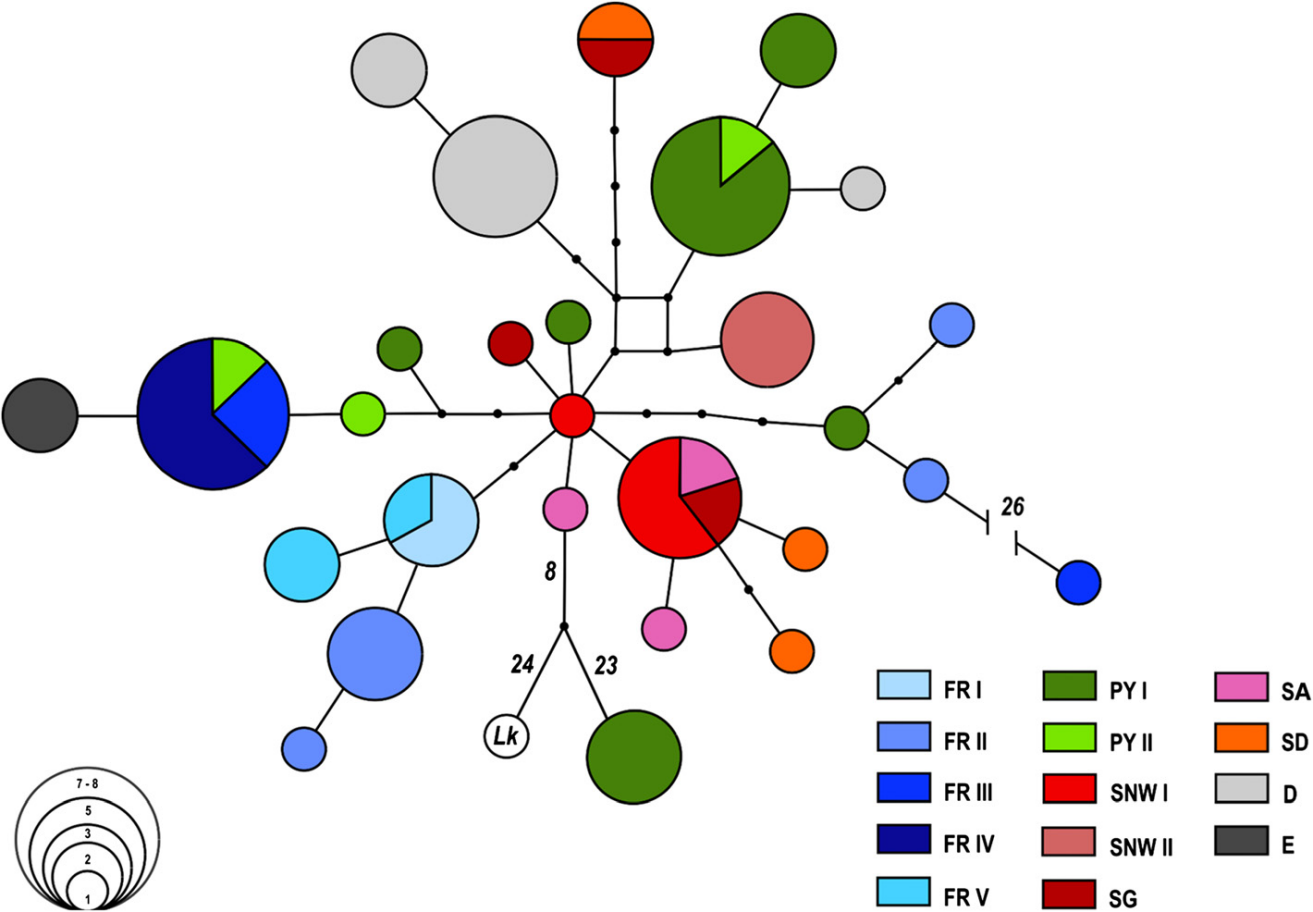
Haplotype network of *L. acervorum* from SW-Europe, Germany (D) and England (E). Populations and geographic regions are labelled by different colours. The size of haplotypes is proportional to the number of individuals (see concentric circles) and each line between haplotypes represents a single mutational step. Included are two extreme divergent haplotypes that differ by 31 (23 + 8) (PY I) and 26 (FR III) mutations and a reference sequence of *Leptothorax kutteri* (*Lk*) that differ by 32 (24 + 8) mutations from the core network, respectively

To evaluate genealogical relationships among sampled populations we reconstructed haplotype networks using the statistical parsimony algorithm as implemented in TCS [47]. The reconstructed network contains two extreme divergent haplotypes, including the three sequences with a 1 bp-deletion and a highly divergent sequence from FR III. These haplotypes are similarly divergent as a reference sequence (same primer pair and PCR conditions used) from the closely related socially parasitic species *Leptothorax kutteri* (Fig. 3). Due to their unclear taxonomic status, these sequences were removed from downstream analysis.

For the quantification of genetic polymorphism we used the following standard diversity indices: number of haplotypes (*h*), number of private haplotypes (*h*_*P*_), number of segregating sites (*S*), haplotype diversity (*H*) and nucleotide diversity (π) for each locality, for each geographic region, and for the whole mtDNA dataset except the extremely divergent haplotypes using DNASP v5.10.01 [48]. We estimated genetic differentiation with and without specifying groups (i.e. geographic regions as defined above), following [49] by calculating *Φ*_*ST*_ – values from mean pairwise differences (*N*_*ST*_) and haplotype frequencies (*G*_*ST*_) in ARLEQUIN. By comparing both statistics of genetic differentiation it is possible to test whether the mtDNA-sequence dataset contains a signal of phylogeographic structure (if *N*_*ST*_ > > *G*_*ST*_) beyond that in haplotype frequencies alone (see [49] for detailed discussion). AMOVA with pairwise comparisons between regions was carried out to test the hypothesis that genetic differentiation is equal or higher within regions (*Φ*_*SC*_) than between regions (*Φ*_*CT*_, regions as defined in the microsatellite data). When both measures are equal, their ratio is expected to be one. Consequently, if their ratio is larger than one, then genetic differentiation is larger within than between regions (which in case of the PY – IB comparison would also mean: no stronger genetic differentiation between social forms). Significance of AMOVA results was evaluated over 10,000 replicates.

#### Demographic history

Mismatch distributions and test statistics for selective neutrality (Tajima’s *D* and Fu’s *F*_*S*_) were calculated for each region and for the whole dataset to measure whether data deviate from expectations of neutral evolution or equilibrium population size. For further details on methods see Additional file 1.

## Results

### Ovary dissections and reproductive skew

Results from ovary dissections are summarized in Table 2. Eighty percent of the inner Iberian colonies (43 out of 54) were functionally monogynous, i.e. only one of several queens that co-occurred in a single nest had elongated ovarioles with traces of previous reproduction (high reproductive skew). The remaining 11 colonies from inner Iberia were either monogynous (*n* = 5) or queenless (*n* = 6). Not a single of the multi-queen colonies collected from various parts of the Pyrenees was functionally monogynous. In contrast, six of 12 Pyrenean colonies had multiple reproductive queens (no. of multi-queen colonies; PY I: 1, PY II: 3, other PY populations: 2), four were monogynous and two were queenless. In accordance with previous studies documenting facultative polygyny in the Pyrenees and other parts of Central and Northern Europe [e.g. [7, 8]) we therefore consider the Pyrenean populations of *L. acervorum* as facultatively polygynous (i.e. low reproductive skew, [8]) and the inner Iberian populations SA and SG as functionally monogynous (Table 2; [8, 11, 20]). Beyond this, we document functional monogyny for the remaining currently known inner Iberian populations of *L. acervorum* (SD, SNW I, SNWII). Further results from ovary dissections are summarized in Additional file 2.

**Table 2:** Reproductive status and skew of Pyrenean (PY) and inner Iberian colonies of *L. acervorum*

Population	*n*	Queenless	Monogyne	Facultative polygyne	Functional monogyne	Reproductive skew
PY	12	2	4	6	0	low
SNW I	7	0	2	0	5	high
SNW II	11	3	0	0	8	high
SG	11	2	1	0	8	high
SD (2009)	15	0	2	0	13	high
SD (2010)	10	1	0	0	9	high
SA^a^	-	-	-	-	-	high

^a^ SA: data from [8, 11, 20]; *n*: number of colonies

### Genetic diversity indices

Significant deviation from HWE after Bonferroni correction for multiple tests (α = 0.00071, see Additional file 2: Table S4) was found in only two of 70 microsatellite locus x population combinations. No evidence of linkage disequilibrium was found for any combination of loci. Thus, we decided to retain all samples and loci in downstream analyses. No clear spatial pattern was found for observed (*H*_*O*_) and expected heterozygosity (*H*_*E*_) (Table 3). The inner Iberian populations (IB: SA, SG, SD, SNW I, SNW II) seemed to have marginally lower values of *k* (range: 6.3 to 9.5), *A*_*P*_ (range: 3 to 14) and *A*_*R*_ (range: 6.0 to 9.0) than the Pyrenean populations (Mann–Whitney U-tests for *k, A*_*P*_ and *A*_*R*_ each: *U* = 10, *N*_1_ = 2, *N*_2_ = 5, *P* < 0.1). However, *H*_*E*_ is generally higher than *H*_*O*_ for all genotyped populations.

**Table 3:** Genetic diversity indices for 10 microsatellite loci in Iberian populations of *L. acervorum*

Region	Population	*n*	*k*	*A*_*P*_	*A*_*R*_	*H*_*O*_	*H*_*E*_
Pyrenees	PY I	15	9.5	11	8.96	0.646	0.803
	PY II	15	8.1	14	7.59	0.665	0.686
Inner Iberia	SNW I	15	7.1	9	6.73	0.571	0.672
	SNW II	15	6.3	4	6.00	0.670	0.687
	SG	15	6.5	3	6.27	0.598	0.741
	SA	15	6.7	8	6.42	0.624	0.722
	SD	15	6.9	4	6.55	0.607	0.710

*n*: no. of individual workers, *k*: no. of alleles, *A*_*P*_: no. of private alleles, *A*_*R*_: allelic richness, *H*_*O*_: observed heterozygosity, *H*_*E*_: expected heterozygosity

Genetic diversity at the mtDNA fragment did not differ strongly between geographic regions (Table 4). Haplotype diversity in the total dataset was generally high (H_D:_ 0.934).

**Table 4:** Genetic diversity indices and neutrality tests for mtDNA sequences of *L. acervorum* in SW- and C-Europe

Region	Population	*n*	*h*	*h*_*P*_	*H*_*D*_	π	*S*	Tajima’s *D*	Fu’s *F*_*S*_
Pyrenees (PY)		14	7	6	0.758	0.0024	14	−0.360	−0.092
	PY I	11	5	4	0.709	0.0020			
	PY II	3	3	1					
Inner Iberian (IB)		15	8	8	0.867	0.0023	15	−0.761	−1.026
	SNW I	3	1	0					
	SNW II	3	1	1					
	SG	3	3	1					
	SA	3	3	2					
	SD	3	3	2					
Southern France (FR)		18	7	6	0.817	0.0030	16	0.227	0.384
	FR I	2	1	0					
	FR II	6	4	4	0.800	0.0031			
	FR III	2	1	0					
	FR IV	5	1	0					
	FR V	3	2	1					
Total	PY + IB + FR	47	21	-	0.934	0.0033	32	−0.889	−6.922*
England	E	2	1	1					
Germany	D	8	3	3	0.607	0.0010			

*n*: no. of sequences, *h*: no. of haplotypes, *h*_*P*_: no. of private haplotypes, *H*_*D*_: haplotype diversity, π: nucleotide diversity, *S*: no. of segregating sites, * *P* < 0.02

*H*_*D*_ for *n* < 6 not shown

### Population structure and phylogeography

Analysis of genealogical relationships among populations (based upon microsatellite data) resulted in an unrooted neighbour-joining tree with nodes supported by bootstrap values between 10 and 96 % (Fig. 2a). The longest internal branch separates the two Pyrenean populations from the remaining Iberian populations. The population from the western Pyrenees (PY II) diverges most among all analysed populations.

Total genetic differentiation (*F*_*ST*_) was low (0.075) and remained moderate even after correction for multiple alleles (*G’*_*ST*_: 0.257, *G”*_*ST*_: 0.265, *D*_est_: 0.205; all values significant with *P* < 0.01). The population from the western Pyrenees (PY II) again showed a higher differentiation than all inner Iberian populations (see Additional file 2).

STRUCTURE runs using all locations strongly supported *K* = 2 genetic clusters (*ΔK*_*2*_: 250.6, *ΔK*_*3*_: 8.6; Additional file 2). With a probability of ≥80 %, 92 % of the individuals from the inner Iberian populations could be assigned to the first cluster, and 95 % of the individuals from the western Pyrenees (PY II) could be assigned to the second cluster (Fig. 2b). In contrast, genotypes from the eastern Pyrenees (PY I) suggested pronounced admixture (Fig. 2b). An analysis without the western Pyrenean (PY II) samples supported four genetic clusters (*K* = 4) within the remaining microsatellite dataset (*ΔK*_*4*_: 5.9, next smaller *ΔK*_*2*_: 5.0, see Additional file 2). Overall, the level of admixture was higher among the remaining populations (Fig. 2c). In particular, PY I, SNW I, and SG showed evidence of high admixture. However, 70 % of the individuals from the remaining Iberian System (SA and SD) could be assigned to a third cluster with a probability ≥80 %, while 67 % of the samples from SNW II could be assigned to a fourth cluster. Tests for isolation by distance (IBD) patterns in the microsatellite data were non-significant for Euclidean (*r*_*xy*_ = 0.010, *P* = 0.5) and log-transformed Euclidean distance (*r*_*xy*_ = 0.083, *P* = 0.4).

The mtDNA-haplotype network has a decentralized structure and shows a short to intermediate expanded genealogy with only limited geographic segregation of haplotypes (i.e. phylogeographic structure, Fig. 3). The only exceptions are two extremely divergent haplotypes from PY I and a nearby population (FR III), which differed from the core network in 31 (23 + 8) and 26 mutations, respectively. In total, the network comprises 28 haplotypes (22 haplotypes without extremely divergent, English, and German haplotypes), of which three are dominant. The first is predominantly distributed in southern France and western Pyrenees (7 + 1 sequences, among them two sequences from FR III), the second from the Pyrenees (6 + 1 sequences from PY I and PY II, respectively) and the third predominantly in inner Iberia (five sequences in total). The remaining French samples formed a second predominantly French haplogroup (Fig. 3).

Significant genetic differentiation (*Φ*_*ST*_ estimated as *G*_*ST*_ and *N*_*ST*_) was observed for the total dataset from Spain and France (extremely divergent haplotypes excluded) and for all pairwise combinations of geographic regions (Table 5, all with *P* < 0.01). The global analysis without regional partitioning of data revealed moderate genetic structure within the whole dataset as well (*Φ*_*ST*_ = 0.498, *P* < 0.001). The data did not reveal strong phylogeographic structure (i.e. *N*_*ST*_ is never larger than *G*_*ST*_) in pairwise comparisons of regions or the whole dataset including all regions (Table 5, and see Fig. 3 for similar results on network structure).

**Table 5:** Results of analysis of molecular variance (AMOVA) by pairwise comparison between regions for *L. acervorum*

Regional pairs	Fixation index			Percentage of variation		
*STR*	*F*_*CT*_	*F*_*SC*_	*F*_*ST*_	*V*_*A*_	*V*_*B*_	*V*_*C*_
IB – PY	0.024 (0.026)	0.068 (0.068)***	0.090 (0.092)***	2.40 (2.58)	6.60 (6.63)	90.99 (90.80)
*mtDNA*	*G*_*CT*_ *(Φ*_*CT*_ *)*	*G*_*SC*_ *(Φ*_*SC*_ *)*	*G*_*ST*_ *(Φ*_*ST*_ *)*	*V*_*A*_	*V*_*B*_	*V*_*C*_
IB – PY	0.107 (0.162)	0.232** (0.231*)	0.314 (0.355)***	10.65 (16.20)	20.76 (19.33)	68.59 (64.47)
PY – FR	0.041 (0.123)	0.378 (0.506)***	0.403 (0.567)***	4.08 (12.30)	36.23 (44.41)	59.69 (43.29)
FR – IB	0.088* (0.186**)	0.459 (0.506)***	0.507 (0.597)***	8.14 (8.75)	35.12 (41.92)	56.74 (49.33)
IB – PY – FR	0.070 (0.154*)	0.365 (0.430)***	0.410*** (0.251**)	7.01 (15.35)	33.97 (36.40)	59.02 (48.25)

Fixation indices were calculated from haplotype frequencies (*G*_*XX*_) and pairwise differences (*Φ*_*XX*_) respectively. Values for null allele corrected microsatellite data (*STR*) in brackets. *V*_*A*_ */Φ*_*CT*_ */F*_*CT*_ *:* among regions relative to the total population, *V*_*B*_ */Φ*_*SC*_ */F*_*SC*_ *:* among local populations within regions, *V*_*C*_ */Φ*_*ST*_ */ F*_*ST*_ *:* among local populations relative to the total population, significance levels: * *P* < 0.05, ** *P* < 0.01, *** *P* < 0.001, asterisks outside brackets refer to both values per fixation index. For a definition of regions see text and Tables 1 & 4

**Fig. 4:**
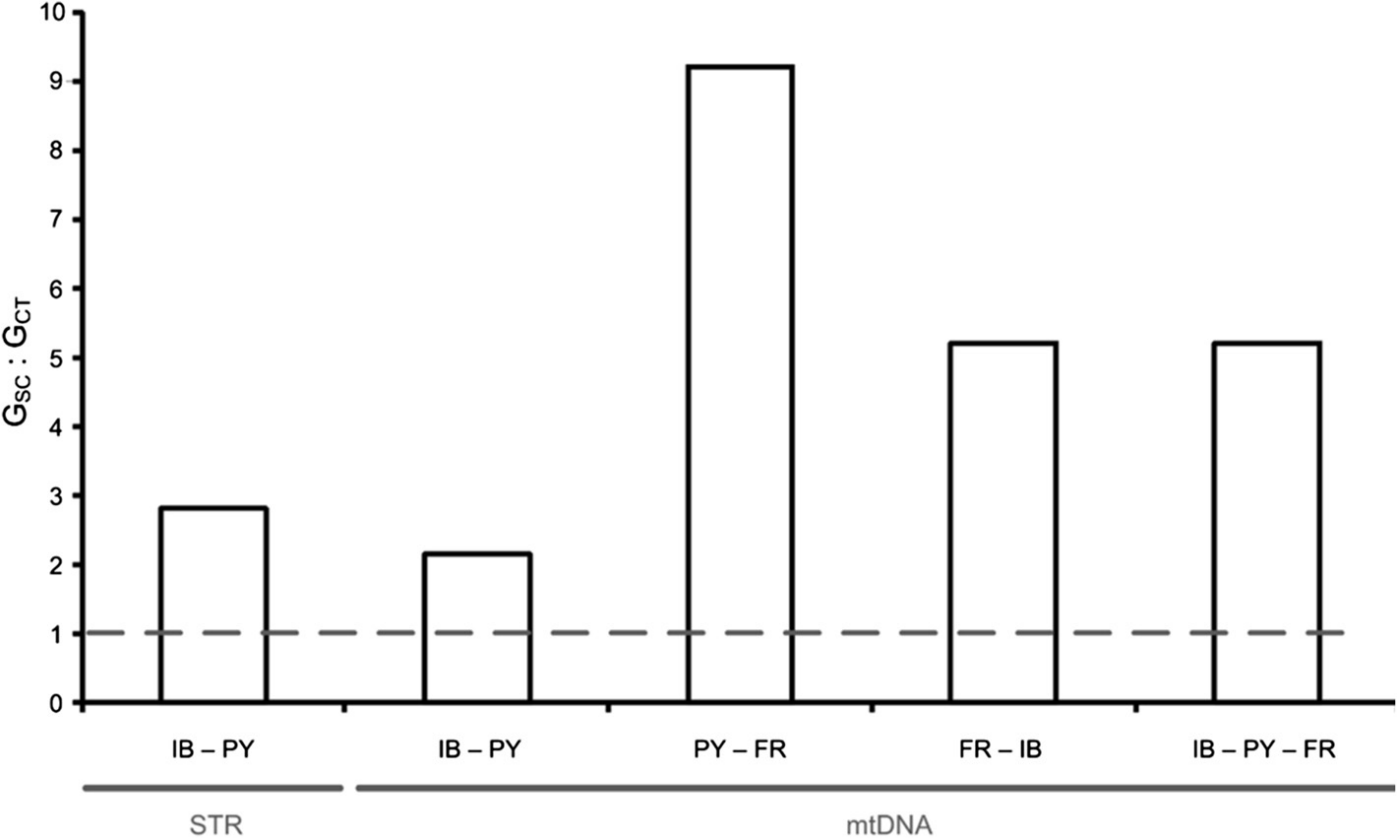
Pairwise comparison of genetic differentiation within versus between geographic regions (given as G_SC_ : G_CT_ ratio and calculated within an AMOVA framework). *Dashed line* indicates 1:1 ratio, above which differentiation within regions is larger than between regions. Comparison on microsatellite data (STR) included the IB – PY regional pair only, while comparison on mtDNA data included all regional pairs. Additionally, a comparison including mtDNA sequences from all regions was conducted (IB – PY – FR). For a definition of regions see text and Tables 1 & 4

In addition, for microsatellite and mtDNA datasets genetic differentiation within regions was higher than genetic differentiation among regions for all combinations of regions, with *Φ*_*SC*_ : *Φ*_*CT*_ ratios > 2 (range: 2.17–9.22, Fig. 4).

### Demographic history

**Fig. 5:**
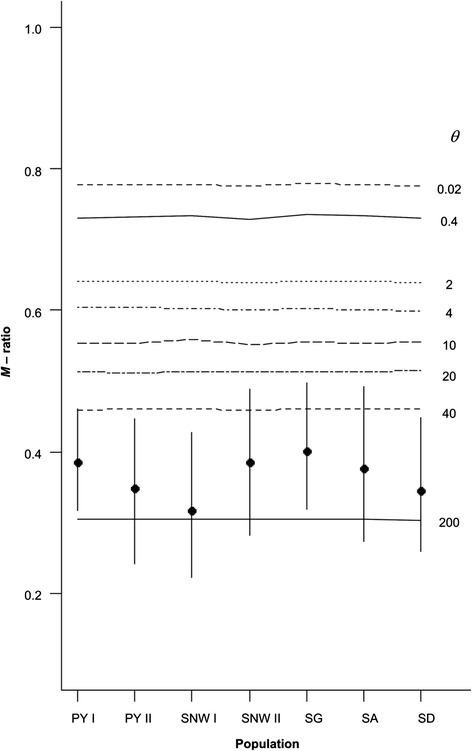
Observed *M*-ratio for each Iberian population (closed circles with error bars indicating 95 % CIs). *Horizontal lines* indicate critical threshold values of *M* (*M*_*C*_) simulated for equilibrium populations at different *θ*-values

Analyses of recent demographic events by summary statistical tests of microsatellite variation provide evidence for demographic bottlenecks. Results from the Wilcoxon test (BOTTLENECK) showed that two inner Iberian populations have a significant excess of heterozygosity only, indicating a recent bottleneck (95 % TPM, SG: *P* = 0.0015; SA: *P* = 0.0093, see Additional file 2). In contrast, *M*-ratio tests suggest that all populations experienced bottlenecks, with observed values well below most critical values for all but the most extreme values of *θ* (i.e. *θ* > 20, corresponding to an effective pre-bottleneck population size of ~50000 individuals, Fig. 5), including the threshold of the empirically derived *M*-value of 0.68.

**Fig. 6:**
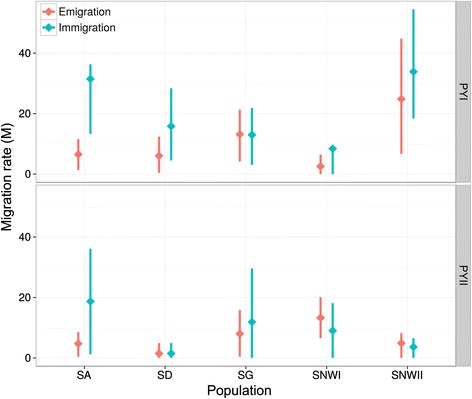
Mean pairwise migration rates for Iberian populations of *L. acervorum* (with error bars indicating 95 % CIs). Rates are given as immigration and emigration separately, with respect to Pyrenees (PY I and II)

Results from MIGRATE-N are summarized in Figs. 1, 6 and Additional file 2. Aim of these analyses was to infer the directionality of migration rates between Iberian and Pyrenean populations and between PY I and PY II. Overall M_NET_ values indicate an asymmetric migration from Iberian populations to the Pyrenees (Fig. 1). None of the pairwise rates of emigration out of the Pyrenees was higher than those of immigration into the Pyrenees (Fig. 6 and Additional file 2). While the mean migration rates (M) and M_NET_ rates between PY II and Iberian populations were low (mean M: 1.47–13.29, M_NET_: 1–1.49; except SA to PY II; M = 18.7, M_NET_ = 3.95), the net migration from Iberian populations to PY I was high (M: 2.56–36.3, M_NET_: 1.01–5.57). The highest mean migration rate was from SA to PY I (mean M = 36.3), approximately two fold higher than between SA and PY II. Differences between migration rates for the Pyrenean populations provide support for higher immigration from Iberian populations to PYI and the relatively isolated nature of PY II. For all sampled populations, Bayesian Skyline plots show a long-term decrease in population sizes starting from various T_BOTTLENECK_. Similar trends of an acute reduction in population sizes were observed for PY II, SNW I and SA (see Additional file 2).

Analyses of historical demographic events using summary statistical tests of mtDNA variation provided no significant evidence for demographic changes or selective events within each geographic region (Table 4).

## Discussion

### Spatial distribution: behavioural polymorphism and genetic structure

In this study, we provide data on the distribution of the two social forms (facultative polygyny vs. functional monogyny) of the ant *Leptothorax acervorum* in the Iberian Peninsula and Pyrenees. We show that colonies are functionally monogynous in all studied inner Iberian mountain ranges, as previously shown for the populations of Sierra de Albarracin (SA) and Sierra de Gúdar (SG) [8, 11, 20]. In contrast, colonies from the Pyrenees are facultatively polygynous, like those from other areas in Central and Northern Europe [7]. This supports previous studies according to which high skew may be an adaptation to difficult queen dispersal in patchy habitats, as commonly found at the margin of a species’ range (see [10, 50] for further discussion).

On a first glance, our population genetic data seem to support an association of genetic differentiation and social organization in *L. acervorum* (e.g. Fig. 2a). However, a more detailed analysis shows stronger genetic differentiation within than between social forms (Fig. 4 & Table 5). In particular, western Pyrenees (PY II) revealed the highest pairwise differentiation to all inner Iberian populations while individuals from eastern Pyrenees (PY I) revealed pronounced admixture from both genetic clusters. Furthermore, haplotype network analysis (mtDNA) failed to find a clear pattern of differentiation between the two social forms.

These results suggest a lack of reproductive isolation between both social forms, contrasting with the stability of social phenotype in the laboratory [20]. Studies with 11 genetic markers might miss genes underlying variation in social structure. For instance, in two ant species queen number is controlled by non-recombining “social chromosomes” [51, 52]. Nevertheless, wherever a genetic basis for social polymorphism has been demonstrated (i.e. via genetic differentiation, phenotypic stability, or genomic rearrangements), different social phenotypes are also associated with mtDNA differentiation (for a literature review see Additional file 2). The lack of mtDNA divergence in *L. acervorum* might therefore speak against a robust genetic basis of the social polymorphism. Furthermore, experimental manipulation of colonies from a low skew population elicited queen antagonism [50] and high skew [53], suggesting that queens can adapt their behaviour to environmental changes. It remains unknown whether queens from functionally monogynous populations behave similarly flexibly, have lost their behavioural plasticity, or have a changed threshold for queen fighting. Finally, phylogenetic analysis showed that functional monogyny evolved convergently in several lineages of the genus *Leptothorax* and thus corroborates skew as a flexible trait that can evolve with environmental changes [54].

### Phylogeography within Iberian Peninsula

Patterns of genetic diversity and genetic structure in *L. acervorum* in inner Iberian mountains match recent studies that revealed substantial geographic complexity in genetic diversity, divergence, and extent of admixture among and within Iberian species (“refugia within refugia hypothesis”; see [55, 56] for reviews). The fragmented Iberian landscape with many mountain ranges and river basins allowed the survival of populations in multiple refugia and facilitated their partial differentiation in allopatry, followed by secondary contact between previously differentiated genomes [56]. *L. acervorum* is a cold-adapted ant species [16, 17] and in the Iberian Peninsula lives mostly in mountainous pinewoods or pine-dominated forests above 1500 m a. s. l. Populations may have co-expanded and -contracted with *Pinus sylvestris*-dominated forests during glacial and interglacial periods. This suggests larger population sizes and higher levels of gene flow among inner Iberian populations during glacial and transitional phases (e.g. early Holocene). In contrast, during the last interglacial populations would have fragmented and declined.

Our molecular analysis supports such a scenario: on a local scale, microsatellites suggest no current gene flow between inner Iberian populations. In addition, the patchy distributions of private alleles (*A*_*P*_) and the occurrence of populations with high vs. low admixture (PY I, SG, SNW I vs. PY II, SNW II, SA, SD) are in accordance with the existence of multiple refugia and varying connectivity among them throughout the late Pleistocene. In contrast, mtDNA data did not reveal a pronounced genetic differentiation among Iberian populations.

The westernmost Cantabrian population (SNW II) provides a notable exception to this pattern. It shows a distinct mtDNA haplotype and forms a distinct genetic cluster in the multi-locus microsatellite data, suggesting a longer period of evolution in isolation. This resembles the situation in the capercaillie (*Tetrao urogallus*), a bird characteristic of Eurasian coniferous forests [57, 58]. Its Cantabrian population inhabits deciduous forests and is the most genetically distinct and depauperate in Europe except for the Pyrenean population, to which it is closely related [59, 60].

Because of the fragmented distribution of *L. acervorum* in the Iberian mountains, we tested for the occurrence of bottlenecks. The mtDNA demographic analyses (summary statistical tests, mismatch distributions, raggedness) did not provide clear evidence for historical changes in effective population sizes. In contrast, *M*-ratios in microsatellites were far lower than the critical *M* value of 0.68 (as derived by [41]) in all populations, including the Pyrenees. When compared to simulated critical *M* values, all seven populations seem to have experienced bottlenecks at values of *θ* (i.e. mutation rate scaled effective population size) that are reasonably large for *L. acervorum* (*θ* = 20–40, corresponding to pre-bottleneck *N*_*e*_ of ~50,000–100,000). In addition, MIGRATE-N analysis confirmed the occurrence of bottlenecks in all seven populations, while excess heterozygosity confirmed recent bottlenecks in only two populations from the Iberian system (SA and SG). The different results between tests can be explained by differences in test procedures and the low power of the heterozygosity excess test due to low sample size or deviations in mutation models (for a detailed discussion see [40, 61] and references therein). We therefore conclude that by the above mentioned properties the heterozygosity excess test is more likely underestimating the number of bottlenecked populations compared to the other used bottleneck test procedures (in particular when bottlenecks did not occur recently). Furthermore, the consistently lower observed than expected heterozygosities (i.e. lower frequency of heterozygote genotypes than expected under Hardy-Weinberg equilibrium) are in agreement with the occurrence of bottlenecks in all inner Iberian and Pyrenean populations. Finally, the occurrence of bottlenecks in the recent past is in accordance with postglacial range contractions or shifts and the current patchy distribution (above 1500 m. s. l.) of *L. acervorum* in the Iberian Peninsula, as indicated by both genetic datasets.

### Relationships of Iberian populations to the adjacent regions (Pyrenees, W-Alps and Central Europe)

In contrast to the clear signal of population structure (microsatellites) among Iberian populations showed here for the first time, mtDNA sequence data did not reveal any spatial-genetic structure. This matches former studies on *L. acervorum* across Europe [18, 20, 62]. The data do not support the existence of a single SW-European refugium but two glacial refugia in France alone. One haplotype, found exclusively in the western Pyrenees (PY II), southernmost Massif Central (FR III) and Mont Ventoux (FR IV), suggests a refugium extending from the lower Rhône valley westward to the foothills and lowlands north of the Pyrenees. A second refugium in the upper Rhône valley or adjacent areas in the north is indicated by a haplogroup found in the remaining French localities (FR I, II, V). Glacial refugia north of the Mediterranean peninsulas have recently been recognized for several species [4, 63–67]. In particular, artic-alpine species show patterns of genetic association similar to *L. acervorum* in southern and Central France (e.g. [3]).

Subsequent to the late glacial (post-10 ka BP), the first French refugium may have started to fragment and *L. acervorum* shifted its range towards more suitable habitats in the surrounding mountains and their foothills, while at the same time populations of the second refugium would have expanded their range at least towards Massif Central and western Alps. A closely related haplotype from England suggests a direct connection to the first French refugium (including two sequences that only differ by one mutation from the main haplotype of the latter, Fig. 3). Analogous scenarios of postglacial range dynamics have been described for other European mountain species (e.g. [3]). For example*,* patterns of genetic structure within and between W-European high mountains of the mountain butterfly *Erebia epiphron* suggest (i) recolonization of the central and western Pyrenees and western Alps from refugial lowlands in between and (ii) of the eastern Pyrenees from the unglaciated foothills south-east and east of it [68].

Similar to the latter scenario, our analysis revealed a differential contribution of the first French refugium and the inner Iberian refugial system to the postglacial recolonization of Pyrenees not just in colonization routes but also in patterns of gene flow by both genetic markers. In contrast to the French refugium, inner Iberian populations share no haplotypes with the Pyrenees (in particular the eastern Pyrenees), while microsatellite data indicate historical gene flow between inner Iberia and eastern Pyrenees, but no or only very restricted gene flow between the western Pyrenees and inner Iberian populations. These patterns suggest that during the early Holocene *L. acervorum* still could maintain gene flow between unglaciated parts of the eastern Pyrenees (PY I) and mountains of the Iberian System (e.g. via a network of suitable habitat patches in the Ebro basin). Subsequently, gene flow gradually broke down in parallel with the increasing aridification and the decline of pinewoods in the Ebro basin during the second half of Holocene [69–71]. The limited gene flow between inner Iberia and W-Pyrenees (PY II) at the same time, can be explained by the stronger influence of oceanic climate and consequently the replacement of pinewoods by broad-leafed tree taxa in the Cantabrian Mountains and W-Pyrenees [70, 72, 73].

## Conclusions

Our study gives important insight into the evolutionary and demographic history of *L. acervorum* in the Iberian Peninsula, the genetic relationship among its two social phenotypes and the recolonization of its Western-Palearctic range from glacial refugia north of the Pyrenees. First, our analyses show a lack of reproductive isolation between both social forms, which indicates that behavioural flexibility rather than a hard-wired genetic basis is underlying variation in social phenotype. Second, we demonstrate the occurrence of substantial genetic structure, which is congruent with the complex Iberian topography and SW-European climate history. Moreover, our results highlight the vulnerability of *L. acervorum* in Iberian mid Mountain ranges to climate change and human induced climatic warming in particular, where the species inhabits suitable microhabitat patches over 1500 m a. s. l. only. A complete picture of the postglacial migration of this Holarctic ant with samples from across its whole range in Eurasia and Northern America as well as new marker sets might help to better understand the dynamics of centre-to-periphery pattern of genetic variability and the history of boreal insect species with high dispersal capacity.

## Additional files

Additional file 1: Additional materials and methods. (DOC 44 kb) Additional file 2: Additional data (including GenBank accession numbers for sequence data). (DOC 795 kb)
