# Supplementary material for: Phylogeography of social polymorphism in a boreo-montane ant
Source: BMC Evol Biol. 2016 Jun 23;16:137. doi: 10.1186/s12862-016-0711-3 (PMC4918132; doi:10.1186/s12862-016-0711-3)
Supplement: Additional file 1: — Additional materials and methods. (DOC 44 kb) [file 12862_2016_711_MOESM1_ESM.doc]

**Additional file 1: Additional materials and methods**

**Ant keeping and ovary dissection**

All queens were killed by freezing at -20°C and their ovaries were subsequently dissected under a binocular microscope using standard methodology (Buschinger & Alloway, 1978). We noted the presence of sperm in the spermatheca, corpora lutea, and mature oocytes and classi­fied ovarian status as described in (Trett*in et* al., 2014) and (Heinz*e et* al., 1992). Reproductive skew in the different populations was estimated by recording the number of colonies with no queen, one queen (monogynous), more than one fertile queen (polygynous) or only one fertile queen among several inseminated, but non-laying female reproductives (functionally monogynous, sensu Buschinger, 1968). For completeness, we added literature data from a fifth Iberian population (SA, Felke & Buschinger, 1999; Gil*l et* al., 2009; Tretti*n et* al., 2011).

**Population structure, gene flow and demographic history (microsatellites)**

The Wilcoxon’s sign rank test (BOTTLENECK 1.2.02) was performed assuming the strict stepwise mutation model (SMM) and the two-phase model (TPM) with probability of single-step mutations set to 95%. The variance for mutation size was set to 12 in both cases as recommended by Pir*y et* al., (1999) and Peer*y et* al., (2012) respectively. Altogether, 110,000 simulations were run.

The *M*-ratio (Garza & Williamson, 2001) is calculated from the number of alleles and the

allelic range per locus, whereby the number of alleles is expected to decline faster than the allelic range during a bottleneck. The mean *M*-ratio per population was calculated in ARLEQUIN. Ninety-five percent confidence intervals (CI) of the *M*-ratio were obtained by bootstrapping over loci for 9,999 replicates using the *boot* package in *R* (R Core Team, 2015). We compared observed *M*-ratios to the upper limits of empirically derived *M*-values after a bottleneck (= 0.68; Garza & Williamson, 2001) and to critical *M* values (*M_c_*) simulated from populations in mutation-drift equilibrium. We used the following parameter settings: eight values of *θ* ranging from 0.02 to 200 [where *θ* = 4*N_e_µ*; *N_e_* is the pre-bottleneck effective population size and *µ* the microsatellite mutation rate, herein assumed to be in the order of 10^-4^ per locus and generation (see Bhargava & Fuentes, 2010 for a review)], the mean size of larger mutations; *δ_g_* = 3.5, and the proportion of multi-step mutations; *p_g_* = 0.2. Both parameters specify how similar the chosen microsatellite mutation model (i.e. herein a 20% TPM) is to a strict single-step SMM. All simulations were run with 10,000 replicates per population and *θ* in the program CRITICAL M (http://swfsc.noaa.gov/textblock.aspx?Division=FED&id=3298).

For the estimation of pairwise migration rates (MIGRATE-N) three replicate runs were conducted, each consisting of a burn-in of 500,000 followed by sampling of 5 million steps. Uniform priors were placed on both the M (m µ^-1^; 0-100) and *θ* (4Neµ; 0-100) parameters. The Brownian model of microsatellite evolution was selected. Relative mutation rates for each locus were estimated by the software itself. Convergence of the runs was checked using the CODA package in R. Apart from *θ* and M, changes of demographic parameters were recorded and inferred from Bayesian Skyline results of MIGRATE-N. Owing to the high number of pairwise migration rates calculated (n=42, 6 per population), net asymmetric migration rate M_NET_ between two populations was estimated by dividing pairwise migration rates by the higher of the two as the numerator (i.e. for migration M_ij_ between i and j locations; net migration, Mij_net_ = Mij _max_ Mij _min_^-1^). The directionality of M_NET_ is determined by the direction of Mij _max_. It is important to note that MIGRATE-N considers shared polymorphisms between two populations as resulting from migration; hence, it calculates mean long-term migration rates and does not distinguish between initial colonization and contemporary gene flow. Our purpose for this analysis was to infer asymmetric migration rates and to identify source and sink populations. Therefore, directionality was inferred only when migration in one direction was at least double of that in the other direction.

**Mitochondrial DNA analyses**

All novel sequences were deposited in GenBank (accession numbers: KU245567-KU245629).

*Demographic history*

After excluding samples from Germany, England and the extremely divergent haplotypes, significance of neutrality tests (Tajima’s *D* and Fu’s *F_S_*) was evaluated over 10,000 replicates in ARLEQUIN. We evaluated the demographic history of *L. acervorum* from SW-Europe by plotting the distribution of pairwise differences between haplotypes (mismatch distribution, Rogers & Harpending, 1992) for each region and for the combined dataset. Afterwards, we compared the fit of observed mismatch distributions to simulated distributions from a sudden expansion model. All calculations were done in ARLEQUIN, using 1,000 replicates to evaluate the significance of results.

**References**

Bhargava, A. & Fuentes, F.F. (2010) Mutational dynamics of microsatellites. *Molecular Biotechnology*, **44**, 250–266.

Buschinger, A. & Alloway, T.M. (1978) Caste polymorphism in *Harpagoxenus canadensis* MR Smith (Hym. Formicidae). *Insectes Sociaux*, **25**, 339–350.

Heinze, J., Lipski, N. & Hölldobler, B. (1992) Reproductive competition in colonies of the ant *Leptothorax gredleri*. *Ethology*, **90**, 265–278.

R Core Team (2015) *R: A Language and Environment for Statistical Computing.* R Foundation for Statistical Computing, Vienna, Austria.

Rogers, A.R. & Harpending, H. (1992) Population growth makes waves in the distribution of pairwise genetic differences. *Molecular Biology and Evolution*, **9**, 552–569.
