# Supplementary material for: Phylogeography of social polymorphism in a boreo-montane ant
Source: BMC Evol Biol. 2016 Jun 23;16:137. doi: 10.1186/s12862-016-0711-3 (PMC4918132; doi:10.1186/s12862-016-0711-3)
Supplement: Additional file 2: — Additional data (including GenBank accession numbers for sequence data). (DOC 795 kb) [file 12862_2016_711_MOESM2_ESM.doc]

**Additional file 2: Additional data and results**

**Ovary dissections and reproductive skew**

Of the 528 female reproductives from 66 *L. acervorum* colonies from four Iberian populations and the Pyrenees, 400 were inseminated and 120 were virgin female sexuals that had shed their wings without mating. Spermathecal content could not be determined in eight individuals. Eighty percent of the Iberian colonies (43 out of 54, Table 2) were functionally monogynous, i.e. they contained only one functional queen plus several mated, but non-laying queens.

**Demographic history**

Distributions of pairwise differences calculated from mtDNA sequences for each region are multimodal and values of raggedness are at least by the factor of 2.6 larger (even if non-significant) than the value for the total dataset (raggedness; FR: 0.067, PY: 0.098 and IB: 0.120; for mismatch distributions see Fig. S3). In contrast, analyses of demographic history for the total dataset suggest a sudden population expansion (mismatch distribution: unimodal, non-significant raggedness coefficient: 0.026 and a significant negative value of Fu’s *F_S_* but no significant value of Tajima’s *D*, Table 4).

**Table S1** Null Allele frequencies calculated with MICRO-CHECKER 2.2.3 using Brookfield method per microsatellite locus and for each population. Populations: eastern Pyrenees (PY I), western Pyrenees (PY II), Cantabrian Mts. I (SNW I), Cantabrian Mts. II (SNW II), Sierra de Gúdar (SG), Sierra de Albarracin (SA) and Sierra de la Demanda (SD).

| Locus | Brookfield 1 | *PY I* |
| --- | --- | --- |
| GA1 | 0.085 |  |
| GA2 | 0.126 |  |
| GT223 | 0.056 |  |
| GT218 | 0.127 |  |
| GT1 | 0.073 |  |
| GT2 | -0.029 |  |
| L18 | 0.001 |  |
| Myrt3 | -0.081 |  |
| 2MS67 | 0.193 |  |
| 2MS46(II) | 0.140 |  |
|  |  | *PY II* |
| GA1 | -0.113 |  |
| GA2 | 0.069 |  |
| GT223 | -0.020 |  |
| GT218 | -0.023 |  |
| GT1 | -0.033 |  |
| GT2 | 0.023 |  |
| L18 | -0.014 |  |
| Myrt3 | 0.050 |  |
| 2MS67 | 0.045 |  |
| 2MS46(II) | 0.006 |  |
|  |  | *SNW I* |
| GA1 | -0.009 |  |
| GA2 | 0.074 |  |
| GT223 | -0.049 |  |
| GT218 | 0.032 |  |
| GT1 | 0.028 |  |
| GT2 | 0.211 |  |
| L18 | 0.114 |  |
| Myrt3 | 0.020 |  |
| 2MS67 | 0.164 |  |
| 2MS46(II) | -0.046 |  |
|  |  | *SNW II* |
| GA1 | -0.054 |  |
| GA2 | -0.017 |  |
| GT223 | -0.095 |  |
| GT218 | -0.056 |  |
| GT1 | 0.137 |  |
| GT2 | 0.105 |  |
| L18 | -0.050 |  |
| Myrt3 | -0.080 |  |
| 2MS67 | 0.082 |  |
| 2MS46(II) | -0.020 |  |
|  |  |  |
| Locus | Brookfield 1 | *SG* |
| GA1 | 0.075 |  |
| GA2 | -0.004 |  |
| GT223 | 0.065 |  |
| GT218 | 0.039 |  |
| GT1 | 0.135 |  |
| GT2 | 0.234 |  |
| L18 | 0.054 |  |
| Myrt3 | -0.028 |  |
| 2MS67 | 0.064 |  |
| 2MS46(II) | 0.046 |  |
|  |  | *SA* |
| GA1 | 0.011 |  |
| GA2 | -0.013 |  |
| GT223 | -0.092 |  |
| GT218 | 0.243 |  |
| GT1 | 0.068 |  |
| GT2 | 0.101 |  |
| L18 | -0.156 |  |
| Myrt3 | 0.041 |  |
| 2MS67 | 0.065 |  |
| 2MS46(II) | 0.147 |  |
|  |  | *SD* |
| GA1 | 0.112 |  |
| GA2 | -0.021 |  |
| GT223 | -0.041 |  |
| GT218 | 0.021 |  |
| GT1 | 0.136 |  |
| GT2 | 0.091 |  |
| L18 | 0.090 |  |
| Myrt3 | -0.036 |  |
| 2MS67 | 0.135 |  |
| 2MS46(II) | -0.022 |  |

**Table S2** *F_ST_* – values for Pyrenean and inner Iberian population pairs of *L. acervorum*, calculated from 10 microsatellite loci.

| Population | PY I | PY II | SNW I | SNW II | SG | SA |
| --- | --- | --- | --- | --- | --- | --- |
| PY II | 0.065 |  |  |  |  |  |
| SNW I | 0.071 | 0.118 |  |  |  |  |
| SNW II | 0.060 | 0.120 | 0.087 |  |  |  |
| SG | 0.039 | 0.128 | 0.086 | 0.068 |  |  |
| SA | 0.049 | 0.113 | 0.050 | 0.078 | 0.044 |  |
| SD | 0.055 | 0.101 | 0.044 | 0.090 | 0.056 | 0.019 |

For details on ID and location of populations see Table 1 and Fig. 1.

**Table S3** *F_ST_* – values for Pyrenean and inner Iberian population pairs of *L. acervorum*, calculated from null allele corrected microsatellite data.

| Population | PY I | PY II | SNW I | SNW II | SG | SA |
| --- | --- | --- | --- | --- | --- | --- |
| PY II | 0.072 |  |  |  |  |  |
| SNW I | 0.072 | 0.121 |  |  |  |  |
| SNW II | 0.062 | 0.119 | 0.085 |  |  |  |
| SG | 0.038 | 0.127 | 0.082 | 0.068 |  |  |
| SA | 0.050 | 0.115 | 0.048 | 0.076 | 0.047 |  |
| SD | 0.056 | 0.101 | 0.041 | 0.089 | 0.057 | 0.019 |

For details on ID and location of populations see Table 1 and Fig. 1.

**Table S4** Test of deviation from Hardy-Weinberg equilibrium (HWE) in microsatellite loci of *L. acervorum*.

| Population | GA1 | GA2 | GT223 | GT218 | GT1 | GT2 | L18 | Myrt3 | 2MS67 | 2MS46II |
| --- | --- | --- | --- | --- | --- | --- | --- | --- | --- | --- |
| PY I | 0.0653 | 0.0064 | 0.0153 | 0.1155 | 0.0022 | 0.7880 | 0.3220 | 0.9347 | 0.0065 | 0.0060 |
| PY II | 0.1096 | 0.0707 | 0.9008 | 0.4676 | 0.4946 | 0.5268 | 0.0433 | 0.0667 | 0.1050 | 0.2653 |
| SNW I | 0.7236 | 0.0290 | 0.8820 | 0.7046 | 0.0229 | 0.0012 | 0.0890 | 0.9079 | 0.0504 | 0.9895 |
| SNW II | 0.8213 | 0.6189 | 0.8817 | 0.2193 | 0.0371 | 0.4012 | 1.0000 | 0.4460 | 0.1585 | 0.7121 |
| SG | 0.3187 | 0.8237 | 0.4870 | 0.6041 | 0.0008 | 0.0062 | 0.6003 | 0.9193 | 0.0897 | 0.2941 |
| SA | 0.8506 | 0.8227 | 0.8385 | **0.0003*** | 0.0262 | 0.3378 | 0.1442 | 0.3199 | 0.6040 | 0.0082 |
| SD | 0.0426 | 0.5906 | 0.1925 | 0.7484 | **0.0000*** | 0.3919 | 0.2967 | 0.5947 | 0.0926 | 0.5423 |

Significant tests indicated by bold type and asterisk. Significance level adjusted for multiple tests by Bonferroni correction (n = 70):

* *α* = 0.00071. For details on population ID see Fig. 1 and Table 1.

**Table S5** Test for bottlenecks in Iberian populations of *L. acervorum* calculated from microsatellite data, using the 95% two-phase model (TPM95) and strict stepwise mutation model (SMM).

| Population | TPM95 | SMM |
| --- | --- | --- |
| PY I | 0,8623 | 0,9033 |
| PY II | 0,9951 | 0,9976 |
| SNW I | 0,9580 | 0,9878 |
| SNW II | 0,9033 | 0,9473 |
| SG | **0,0015*** | **0,0034*** |
| SA | **0,0093*** | **0,0161*** |
| SD | 0,7539 | 0,8389 |

Significant tests indicated by bold type and asterisk. For details on population ID see Fig. 1 and

Table 1.

**Table S6** MIGRATE-N results for mean migration (for im- and emigration) and net migration

rates (Mnet) between Iberian populations.

| From | To | Mean (M) | 95% low* | 95% up* | Rate |
| --- | --- | --- | --- | --- | --- |
| PYI | SNWI | 2.56 | 0 | 6.40 | Emigration |
| PYI | SNWII | 24.81 | 6.67 | 44.80 | Emigration |
| PYI | SG | 13.15 | 4.13 | 21.33 | Emigration |
| PYI | SA | 6.52 | 1.33 | 11.60 | Emigration |
| PYI | SD | 6.06 | 0.40 | 12.40 | Emigration |
| PYII | SNWI | 13.29 | 6.53 | 20.13 | Emigration |
| PYII | SNWII | 4.88 | 0 | 8.27 | Emigration |
| PYII | SG | 7.99 | 0.40 | 15.87 | Emigration |
| PYII | SA | 4.74 | 0.40 | 8.53 | Emigration |
| PYII | SD | 1.46 | 0 | 4.93 | Emigration |
| PYI | SNWI | 8.42 | 0 | 9.33 | Immigration |
| PYI | SNWII | 33.86 | 18.4 | 54.53 | Immigration |
| PYI | SG | 12.97 | 3.07 | 21.87 | Immigration |
| PYI | SA | 36.30 | 13.33 | 31.47 | Immigration |
| PYI | SD | 15.84 | 4.53 | 28.40 | Immigration |
| PYII | SNWI | 9.02 | 0 | 18.13 | Immigration |
| PYII | SNWII | 3.64 | 0 | 6.53 | Immigration |
| PYII | SG | 11.88 | 0 | 29.60 | Immigration |
| PYII | SA | 18.70 | 1.20 | 36.13 | Immigration |
| PYII | SD | 1.47 | 0 | 4.93 | Immigration |
| Between | Population |  |  |  |  |
| PYI | SNWI | 3.29 | - | - | Mnet |
| PYI | SNWII | 1.36 | - | - | Mnet |
| PYI | SG | 1.01 | - | - | Mnet |
| PYI | SA | 5.57 | - | - | Mnet |
| PYI | SD | 2.61 | - | - | Mnet |
| PYII | SNWI | 1.47 | - | - | Mnet |
| PYII | SNWII | 1.34 | - | - | Mnet |
| PYII | SG | 1.49 | - | - | Mnet |
| PYII | SA | 3.95 | - | - | Mnet |
| PYII | SD | 1.01 | - | - | Mnet |

* Lower and upper bound of 95% Highest Posterior Density interval.

**Table S7** Comparative literature review on social polymorphism in Hymenoptera.

| Species | Type of social  polymorphism | Genetic differentiation  between forms | | Genetic  basis | Phenotypic  plasticity | Spatial  distribution | Reference |
| --- | --- | --- | --- | --- | --- | --- | --- |
|  |  | mtDNA | ncDNA |  |  |  |  |
| *Solenopsis invicta* | soc. structure (Q-no.) | yes | partial | gen diff,  gec ream | no | sympatric | [1-3] |
| *Formica selysi* | soc. structure (Q-no.) | n/a | no | "social chromosome" | no | sympatric | [4-6] |
| *Formica fusca* | soc. structure (Q-no.) | n/a | no | suggested  (field obs) | no | sympatric | [7] |
| *Formica*  *truncorum* | soc. structure (Q-no.) | yes | yes | gen diff | no | sympatric | [8] |
| *Veromessor pergandei* | colony foundress (Q-no.) | n/a | no | suggested  (lab obs) | n/a | para-/ allopatric | [9] |
| *Pogonomyrmex californicus* | colony foundress (Q-no.) | n/a | n/a | suggested  (lab obs) | n/a | para-/ allopatric | [10] |
| *Leptothorax acervorum* | soc. structure (Q-no.)* | no | no | suggested  (lab obs, HS) | yes  (lab exp,LS) | para-/ allopatric | [11-13] |
| *Halictus rubicundus*  (sweat bee) | solitary-social transition | no | no | no | yes  (field exp) | para-/ allopatric | [14, 15] |

* functional monogyny vs. facultative polygyny, gen diff: genetic differentiation, gec ream: genomic rearrangement, obs: observation, exp: experiment,

HS & LS: high & low skew populations.

**References**

[1] Shoemaker, D.D. & Ross, K.G. (1996) Effects of social organization on gene ﬂow in the ﬁre ant *Solenopsis invicta*. *Nature*, **383**, 613-616.

[2] Gotzek, D. & Ross, K.G. (2007) Genetic regulation of colony social organization in fire ants: an integrative overview. *The Quarterly Review of Biology*, **82**, 201-266.

[3] Wang, J., Wurm, Y., Nipitwattanaphon, M., Riba-Grognuz, O., Huang, Y.-C., Shoemaker, D. & Keller, L. (2013) A Y-like social chromosome causes alternative colony organization in fire ants. *Nature*, **493**, 664-668.

[4] Chapuisat, M., Bocherens, S. & Rosset, H. (2004) Variable queen number in ant colonies: no impact on queen turnover, inbreeding, and population genetic differentiation in the ant *Formica selysi*. *Evolution*, **58**, 1064-1072.

[5] Purcell, J. & Chapuisat, M. (2013) Bidirectional shifts in colony queen number in a socially polymorphic ant population. *Evolution*, **67**, 1169-1180.

[6] Purcell, J., Brelsford, A., Wurm, Y., Perrin, N. & Chapuisat, M. (2014) Convergent genetic architecture underlies social organization in ants. *Current Biology*, **24**, 2728-2732.

[7] Bargum, K., Helanterä, H. & Sundström, L. (2007) Genetic population structure, queen supersedure and social polymorphism in a social Hymenoptera. *Journal of Evolutionary Biology*, **20**, 1351-1360.

[8] Gyllenstrand, N., Seppä, P. & Pamilo, P. (2005) Restricted gene ﬂow between two social forms in the ant *Formica truncorum*. *Journal of Evolutionary Biology*, **18**, 978-984.

[9] Helms, K.R. & Helms Cahan, S. (2012) Large scale regional variation in cooperation and conflict among queens of the desert ant *Messor pergandei*. *Animal Behaviour*, **84**, 499-507.

[10] Overson, R., Gadau, J., Clark, R.M., Pratt, S.C. & Fewell, J.H. (2014) Behavioral transitions with the evolution of cooperative nest founding by harvester ant queens. *Behavioral Ecology and Sociobiology*, **68**, 21-30.

[11] Trettin, J., Haubner, M., Buschinger, A. & Heinze, J. (2011) Queen dominance and worker policing control reproduction in a threatened ant. *BMC Ecology*, **11**, 21.

[12] Trettin, J., Seyferth, T. & Heinze, J. (2014) Behavioral Plasticity in Ant Queens: Environmental Manipulation Induces Aggression among Normally Peaceful Queens in the Socially Polymorphic Ant *Leptothorax acervorum*. *PloS One*, **9**, e95153.

[13] Gill, R.J., Arce, A., Keller, L. & Hammond, R.L. (2009) Polymorphic social organization in an ant. *Proceedings of the Royal Society of London B: Biological Sciences*, **276**, 4423-4431.

[14] Soro, A., Field, J., Bridge, C., Cardinal, S.C. & Paxton, R.J. (2010) Genetic differentiation across the social transition in a socially polymorphic sweat bee, *Halictus rubicundus*. *Molecular Ecology*, **19**, 3351-3363.

[15] Field, J., Paxton, R.J., Soro, A. & Bridge, C. (2010) Cryptic plasticity underlies a major evolutionary transition. *Current Biology*, **20**, 2028-2031.


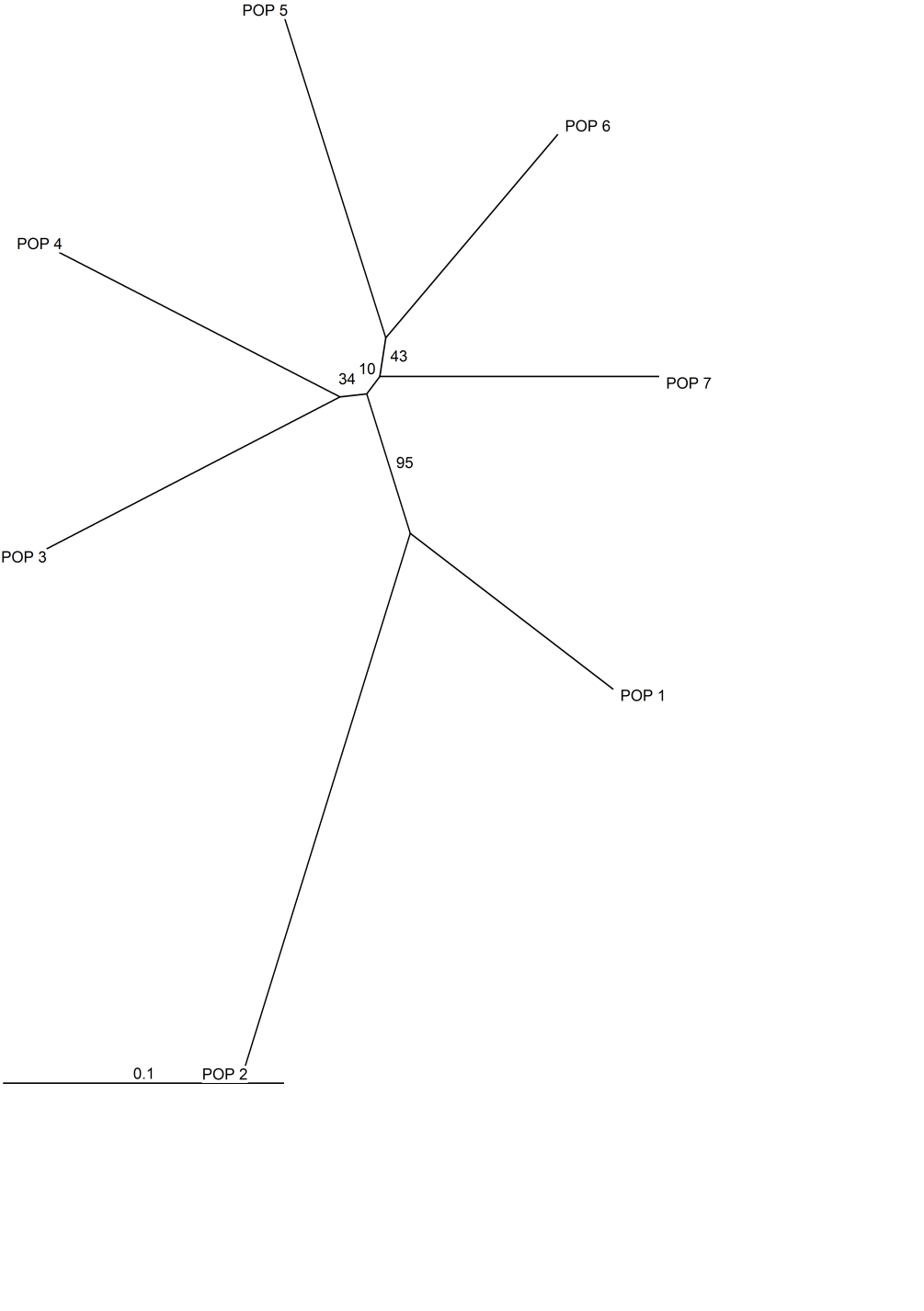


**Fig. S1** Unrooted neighbour-joining tree (for null allele corrected microsatellite data) using Nei's D_A_ distance, with bootstrap values given as numbers close to nodes. POP 1: *PY I*, POP 2: *PY II*, POP 3: *SNW I*, POP 4: *SNW II*, POP 5: *SG*, POP 6: *SA*, POP 7: *SD*. For population's ID and location see Table 1 and Fig. 1.


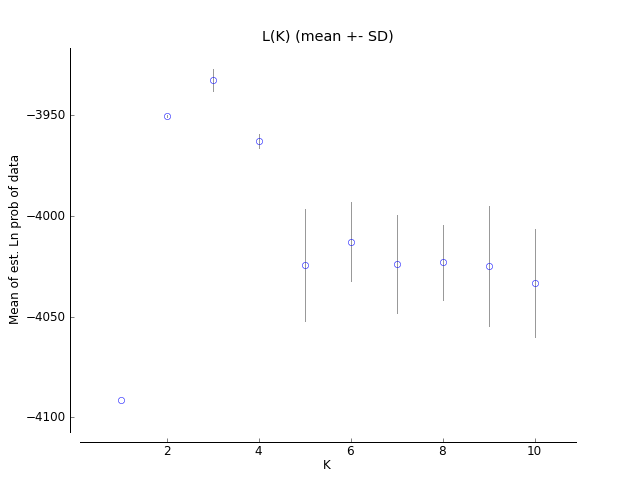

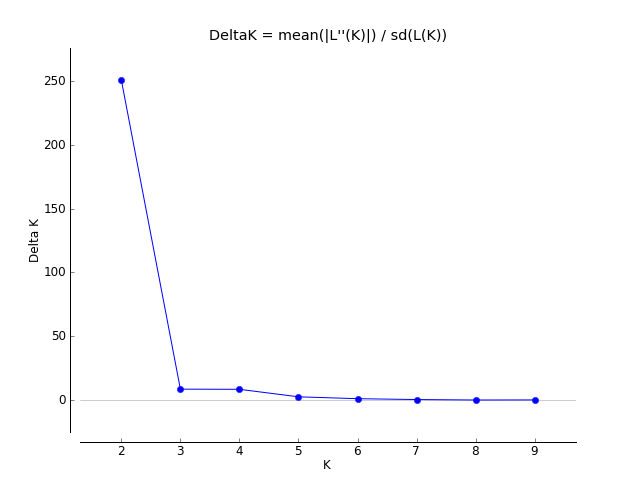

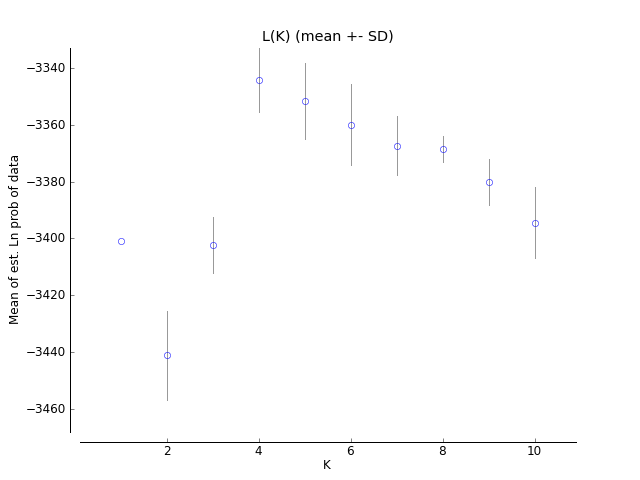

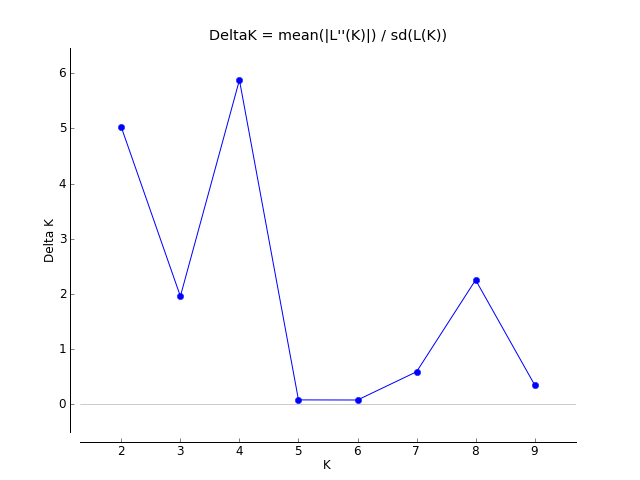


**(A)**

**(B)**

**Fig. S2** Estimated Ln probability of data and ΔK – values for (A) the 1^st^ (all locations included) and (B) 2^nd^ STRUCTURE analysis (PY II removed).

**(IB-PY-FR)**


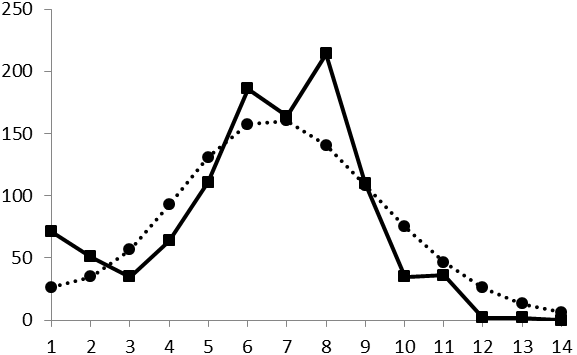

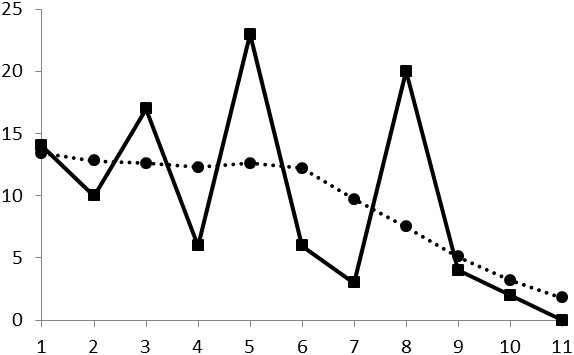

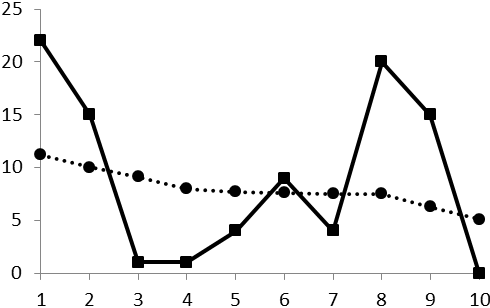

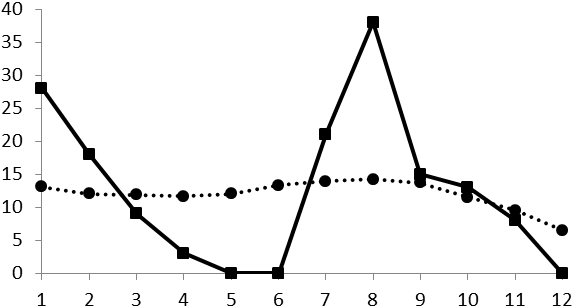


**(IB)**

**(FR)**

**(PY)**

**Fig. S3** Distribution of pairwise differences between mtDNA haplotypes for the total dataset (IB-PY-FR), inner Iberia (IB), Pyrenees (PY) and France (FR). Solid line: Observed frequency, Dashed line: expected frequency (mean of 1000 replicates).


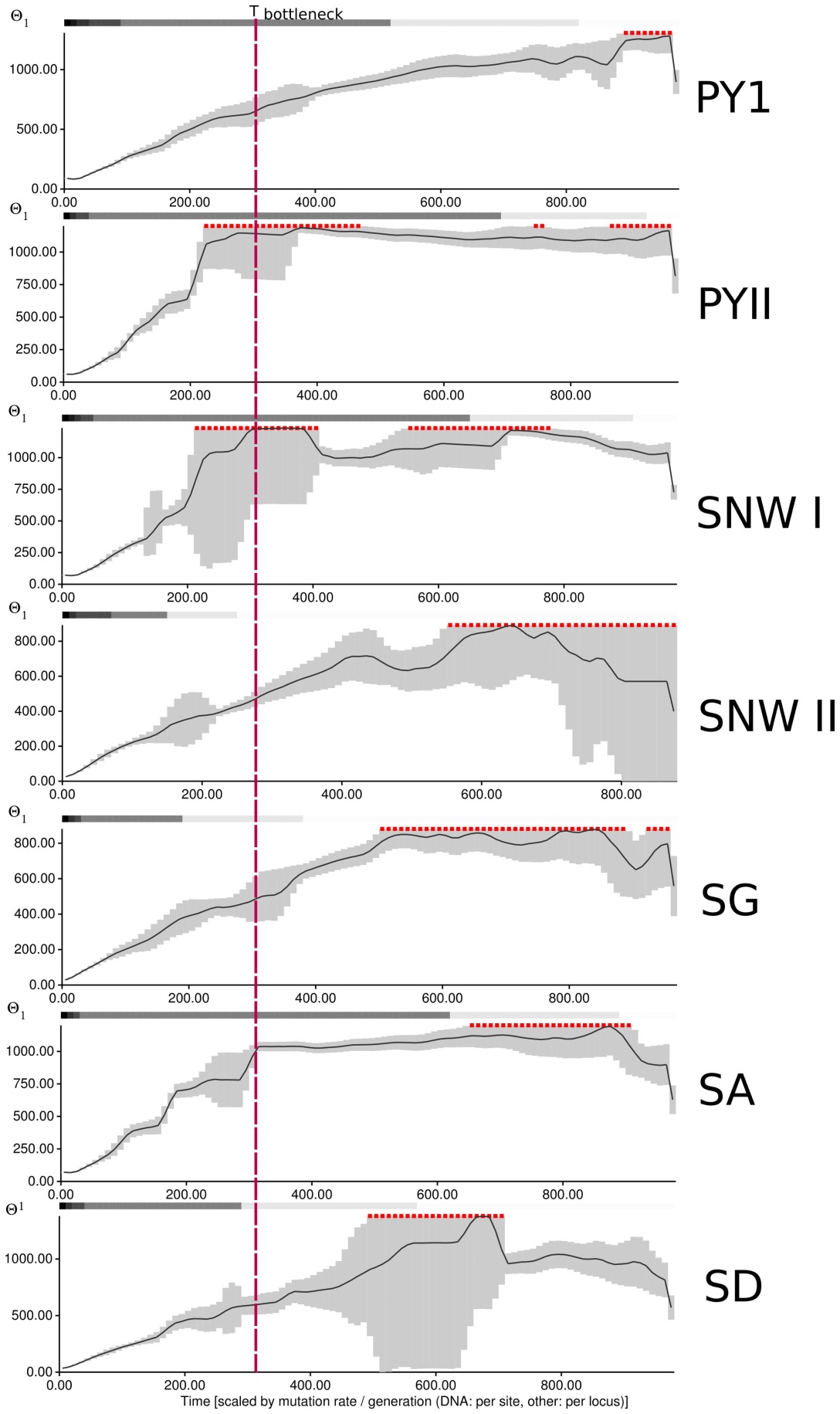


**Fig. S4** Bayesian Skyline plots (estimated from microsatellite data) show a long-term decrease in population sizes (Θ) starting from various T_BOTTLENECK_. Dashed line marks an event of acute population-size reduction experienced by PY II, SNW I and SA. For population's ID and location see Table 1 and Fig. 1.
